# Catalytic Upgrading of Biomass-Gasification Mixtures Using Ni-Fe/MgAl_2_O_4_ as a Bifunctional Catalyst

**DOI:** 10.1021/acs.energyfuels.2c01452

**Published:** 2022-07-18

**Authors:** Pilar Tarifa, Tomás Ramirez Reina, Miriam González-Castaño, Harvey Arellano-García

**Affiliations:** † Department of Process and Plant Technology, Brandenburg University of Technology (BTU) Cottbus-Senftenberg, Platz der Deutschen 1, 03046 Cottbus, Germany; ‡ Department of Chemical and Process Engineering, University of Surrey, Guildford GU2 7XH, United Kingdom; § Department of Inorganic Chemistry and Materials Sciences Institute, University of Seville-CSIC, 41092 Seville, Spain

## Abstract

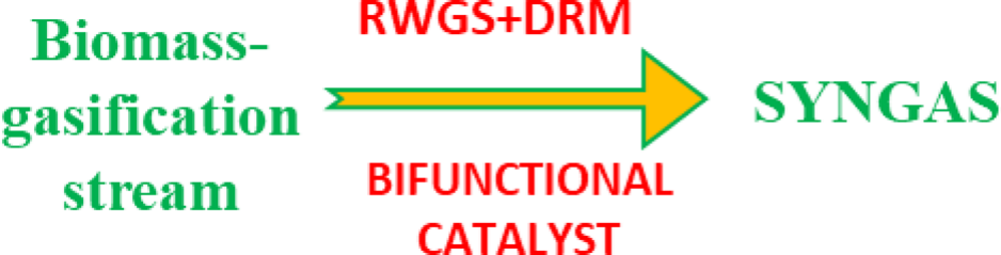

Biomass gasification streams typically contain a mixture of CO, H_2_, CH_4_, and CO_2_ as the majority components and frequently require conditioning for downstream processes. Herein, we investigate the catalytic upgrading of surrogate biomass gasifiers through the generation of syngas. Seeking a bifunctional system capable of converting CO_2_ and CH_4_ to CO, a reverse water gas shift (RWGS) catalyst based on Fe/MgAl_2_O_4_ was decorated with an increasing content of Ni metal and evaluated for producing syngas using different feedstock compositions. This approach proved efficient for gas upgrading, and the incorporation of adequate Ni content increased the CO content by promoting the RWGS and dry reforming of methane (DRM) reactions. The larger CO productivity attained at high temperatures was intimately associated with the generation of FeNi_3_ alloys. Among the catalysts’ series, Ni-rich catalysts favored the CO productivity in the presence of CH_4_, but important carbon deposition processes were noticed. On the contrary, 2Ni-Fe/MgAl_2_O_4_ resulted in a competitive and cost-effective system delivering large amounts of CO with almost no coke deposits. Overall, the incorporation of a suitable realistic application for valorization of variable composition of biomass-gasification derived mixtures obtaining a syngas-rich stream thus opens new routes for biosyngas production and upgrading.

## 1 Introduction

In transition into renewable energies, biomass has received significant attention since it is a promising source for power generation as well as chemical production. It can be processed by different routes (biochemical or thermochemical) with gasification being the most efficient route for power generation (H_2_ and syngas) and easier scalability.^1^ This route is a complex process in which biomass decomposition involves numerous reactions leading to a fairly heterogeneous bioproducer gas. Although the product distribution depends on several variables such as biomass composition or the gasifying agent employed, the main gas components are H_2_, CO, CO_2_, H_2_O, N_2_, and CH_4_.^2−4^ Among them, air is widely used as a gasifying agent given its availability. However, air introduces large amounts of N_2_ which dilutes syngas concentration reducing the calorific value of biosyngas. In this sense, running the gasifier with pure O_2_ solves this issue, although it also increases remarkably the operating costs due to pure O_2_ production typically achieved through energy-intensive cryogenic distillation. On the other hand, H_2_O and CO_2_ are also employed as gasifying agents leading to higher H_2_ and CO concentrations in the gasification products.^3,5−8^ To sum up, according to the literature, the approximate composition of a producer gas obtained with different gasifying agents is depicted in Table 1.

**Table 1:** Producer Gas Composition from Biomass Gasification Using Different Gasifying Agents

gasifying agent	CO_2_ (vol %)	CO (vol %)	H_2_ (vol %)	CH_4_ (vol %)	N_2_ (vol %)	ref
air	10–18	5–28	3–13	0–7	40–50	(9, 10)
O_2_	25–40	20–30	20–30	5–10	0–1	(11)
H_2_O	8–25	20–40	30–50	6–15	0–1	(11, 12)
CO_2_	40–57	20–40	15–18	18–20		(13, 14)

Although this gas can be applied directly to heat, power generation,^15^ or fuel cells in the case of a steam gasification stream,^16^ targeting syngas or H_2_ as end products is more appealing given their direct application and versatility for fuel and chemical production.^12,17^ For H_2_-rich streams, the producer gas reacts by water gas shift (WGS) and steam reforming of methane (SRM) reactions in which, essentially, methane and CO end up as CO_2_ which is subsequently scrubbed by adsorption methods.^18^ Thus, this route implies the production of CO_2_ as a byproduct and expensive downstream CO_2_ capture and storage technologies. Therefore, in this work, we propose the biomass-gasification derived mixture upgrading via reverse water gas shift (RWGS) and dry reforming of methane (DRM) reactions to optimize the overall syngas production with the minimum carbon loss resulting in a syngas-rich feedstock.

This approach aims to optimize carbon uptake from initial biomass/biowaste, and it necessarily requires a custom-made catalyst to undertake both RWGS and DRM. Catalysts based on Pd, Cu, Ni, Fe, and Pt metals are widely proposed as active systems for the RWGS reaction. Within moderate temperature windows, Cu and Fe show higher CO selectivity, while Ni metal describes higher methanation rates.^19^ Moreover, the characteristic poor thermal stability of Cu-based catalysts, often improved through the incorporation of textural promoters with higher melting points and lower agglomeration tendencies,^20^ remark Fe as the RWGS active phase. On the other hand, for the DRM reaction, noble metals such as Ru, Rh, Pt, Ni, Ir, or Pd are very active with low carbon depositions. Despite the great activity and coke resistance displayed by Ru and Rh, their price limits their use and tilts the balance toward the application of Ni metal.^21^

Herein, Fe and Ni metals were selected for the catalyst formulation due to its suitable activity in RWGS and DRM reactions and relatively low cost.^22^ Indeed, bimetallic Ni-Fe systems have been widely studied for DRM^23−27^ and also proven effective in the RWGS.^28^ For instance, de Lima et al.^26^ demonstrate that Fe improves the lifetime of the catalyst. Similarly, investigations conducted by Theofanidis et al.^27^ over FeNiMgO catalysts associated the enhanced resiliencies against carbon deposits with the incorporation of Fe oxide. On the other hand, basic or redox materials can also improve the activity and selectivity of Ni- and Fe-based catalysts. For instance, the oxygen vacancies introduced by CeO_2_,^29^ MoO_3_,^30^ TiO_2_,^31^ or Nb_2_O_5_^32^ among others enhance the CO_2_ adsorption and dissociation. Moreover, MgO forms a solid solution which increases thermal stability by constricting the metal sintering.^21^ Another catalytic material industrially applied due to its stability at high temperatures is MgAl_2_O_4_.^21,33,34^ The basic character and fair specific surface areas demonstrated by MgAl_2_O_4_ supported catalysts enable the achievement of long-life active systems and account for its relevance in several CO_2_ conversion reactions like RWGS,^35^ DRM,^36^ or methanation.^37^

This work investigates the generation of syngas from biomass-gasification derived feedstocks over Ni-Fe catalysts supported on MgAl_2_O_4_ spinel. The major focus of this research was developing a bifunctional catalyst capable of converting CO_2_ and CH_4_ simultaneously via RWGS and DRM reactions. In practice though, the presence of CO should be considered since it constitutes an important reactant (ca. 28% when air is used as a gasifying agent), and lower CO_2_ conversions can be expected. Due to the high CO_2_:CH_4_ ratios found in biomass-gasification derived feedstocks, high Fe/Ni ratios were selected in order to promote preferably the RWGS reaction. Thus, the employed strategy used the Fe/MgAl_2_O_4_ catalyst, an active system toward the RWGS reaction as the starting point. For obtaining a bifunctional RWGS-DRM catalyst, the Fe-rich system was decorated with different amounts of Ni metal. The (*X* wt %) Ni – (30 wt %)Fe/MgAl_2_O_4_ (*X* = 2, 5, 10) catalysts’ series was characterized and evaluated under CO_2_:H_2_:CH_4_ reaction atmospheres. The improved catalytic performances regarding activity and stability exhibited by the Ni-Fe/MgAl_2_O_4_ catalyst with low Ni content thereby favored syngas production from biomass-gasification derived feedstock.

## 2 Experimental Section

### 2.1 Synthesis of the Catalysts

The samples were prepared by successive wet impregnation. First, the support (Mg/Al_2_O_3_) was prepared, and afterward, it was impregnated to obtain a catalyst following the procedure described in Alvarez et al.^38^ Briefly, the Mg precursor (Mg(NO_3_)_2_·6H_2_O from Sigma Aldrich) was dissolved in ethanol in order to obtain 10 wt % Mg. Afterward, commercial Al_2_O_3_ spheres (from Sasol Scca 1.8/210) were gridded and sieved between 100 and 200 μm and added to the solution. After 30 min of stirring, the solvent was removed by a rotatory evaporator at 60 °C. Finally, the solid was dried for 12 h at 60 °C and calcined for 12 h at 850 °C heating at 10 °C/min. The support was called MA.

Likewise, the catalysts were synthesized by wet impregnation of the MA. In this case, Fe and Ni precursors (Ni(NO_3_)_2_·6H_2_O from Alfa Aesar and Fe(NO_3_)_3_·9H_2_O from Thermoscientific) were simultaneously impregnated fixing the Fe content at 30 wt % and varying the Ni content from 10 wt % to 2 wt %. In addition, a Ni/Mg-Al_2_O_3_ catalyst with 10 wt % Ni was synthesized as a comparison. After 30 min of stirring, the solvent was removed by a rotatory evaporator and dried at 70 °C for 12 h. Finally, the dried solid was calcined at 500 °C for 3 h of heating at 10 °C/min. The catalysts were labeled as Fe/MA, 2Ni-Fe/MA, 5Ni-Fe/MA, 10Ni-Fe/MA (being 2 wt %, 5 wt %, and 10 wt % of Ni, respectively), and Ni/MA.

### 2.2 Characterization Techniques

The chemical composition of the samples was measured by inductively coupled plasma mass spectrometry (ICP-MS) with Thermo Scientific equipment. The structural compositions (X-Ray Diffraction data, XRD) were obtained by a D2 Phaser diffractometer instrument (from Bruker) equipped with a Cu Kα radiation source (40 mA, 45 kV). XRD measurements were carried out over a 10–70° 2θ range of using a step time of 0.35 s and a size of 0.02°. Crystal sizes (CS) were calculated through the Scherrer equation.

The reducibility of the samples was evaluated by Temperature Programme Reduction (H_2_-TPR) with ChemBet equipment from Anton Paar. 100 mg of the sample was placed in a U-tube reactor. The sample was heated from room temperature up to 900 °C at 10 °C/min using 10% of H_2_ balanced with N_2_. The signal was recorded by a TCD detector previously calibrated with CuO (99.999% purity from Merck KGaA).

The oxygen exchange capacity (OEC) was measured by thermogravimetric analysis with TGA-Ste equipment. 20 mg of the sample was placed into a 40-μL crucible. The sample was *in situ* reduced with a heating rate of 20 °C/min up to 700 °C for 30 min feeding 4% of H_2_ (100 mL/min of total flow). After temperature stabilization at 500 °C, 10 oxidation–reduction cycles were carried out using 4% of CO_2_ and 4% of H_2_, respectively. The sample weight was followed continuously during all of the experiment. The OEC was calculated as the difference between the initial and final weight of each step.

### 2.3 Catalytic Activity

The catalytic activity was evaluated with homemade equipment outfitted with calibrated mass flow controllers (Aalborg) and a Hastelloy tubular reactor (8 mm of inner diameter) coupled with its corresponding furnace equipped with two K-type thermocouples placed in the furnace and inside the reactor. The thermocouple inside the reactor (in contact with the bed catalyst) controlled and monitored the reaction temperature (PID Eng&Tech, Micromeritics). The catalysts were tested using 200 mg diluted with SiC (0.75 cm^3^) under CO_2_:H_2_:CH_4_ mixtures at different reaction temperatures. Prior to the reaction, the catalyst was reduced by being heated up to 700 °C at 10 °C/min for 30 min using 40% of H_2_ balanced with N_2_ (100 mL/min of total flow). Afterward, the reaction was carried out between 400 and 700 °C feeding 15% of CO_2_ in all cases and varying H_2_ between 0% and 60% along with CH_4_ from 0% to 15% (labeled as the CO_2_:H_2_:CH_4_ volume ratio) obtaining a WHSV of 30 Lg^–1^ h^–1^. The composition of the exhausted gases was analyzed by an ABB analyzer, equipped with Uras 26 and Caldos 25 analyzers, and the total flow was measured by a bubble flowmeter. The catalysts’ performance exhibited by the samples was evaluated using the data recorded for the samples after 30 min. The CO productivity and carbon deposition were calculated with eq 1 and eq 2, where *F*_*i*,in_ is the *i* (CO_2_, CH_4_, or CO) specie in the feed, *F*_out_ is the total flow, and *y*_*i*,out_ is the percentage of *i* specie in exhausted gases.
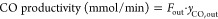
1
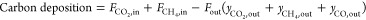
2

## 3 Results and Discussion

### 3.1 Composition of the Catalysts

**Figure 1:**
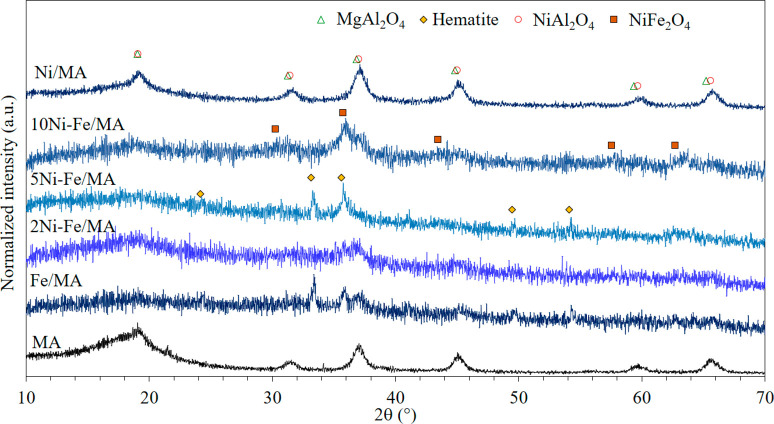
XRD diffractograms of calcined samples.

The chemical composition was measured by ICP, and the results are displayed in Table 2. All calcined catalysts showed weight percentages similar to the nominal ones (30 wt % of Fe and 2 wt %, 5 wt %, and 10 wt % of Ni) evidencing catalysts’ successful preparation. In addition, the structural composition of the calcined samples was analyzed by XRD and illustrated in Figure 1. All samples showed peaks located at 19°, 31°, 36°, 44°, 59°, and 65° which are attributed to MgAl_2_O_4_ spinel, clearly identified in the MA diffractogram. Particularly, the last reflections (59° and 65°) shift to a larger angle in the case of Ni/MA suggesting the formation of NiAl_2_O_4_ spinel. Indeed, the absence of NiO could indicate that the whole Ni was incorporated into the spinel lattice or that NiO is well dispersed presenting a very small crystal size not detectable by XRD. Moreover, in addition to the support signal, two peaks at 33° and 35° are found in the Fe/MA catalyst which corresponds to the hematite phase (Fe_2_O_3_). As the Ni amount increases (Fe/Ni ratio decreases), the peak located at 35° becomes more intense with respect to the 33° peak indicating the formation of Ni-Fe spinel.^39^ Actually, the absence of NiO peaks indicates that Ni was incorporated into the Ni-Fe spinel lattice being the main phase.

**Table 2:** Composition of the Catalysts, Crystal Size, and Reducibility

sample	Ni weight^a^ (%)	Fe weight^a^ (%)	Fe/Ni ratio (-)	CS_Ni-Fe alloy_^b^ (nm)	H_2_ consumption (mmol/g_sample_)
Mg/Al_2_O_3_ (MA)					0
Fe/MA		31			1.45
2Ni-Fe/MA	3	32	11	14	1.94
5Ni-Fe/MA	6	34	6	23	2.19
10Ni-Fe/MA	11	29	3	25	2.22
Ni/MA	13				0.58

a Weight percentages obtained by ICP measurements.

b Calculated through the Scherrer equation applied to the peak located at 43°.

**Figure 2:**
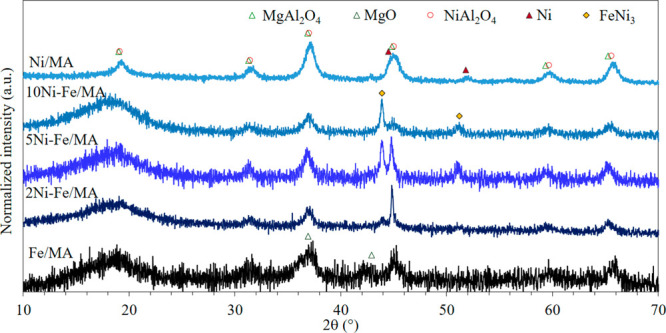
XRD diffractograms of reduced catalysts.

Moreover, the XRD diffractograms acquired from the reduced catalysts are collected in Figure 2. In comparison with calcined samples, the absence of metal oxide phases underlined the constitution of reduced metal phases at 700 °C. In the case of Ni/MA, two peaks appear at 44.5° and 51.8° corresponding to metallic Ni. In Fe/MA, an additional peak located at 42° and attributed to MgO is observed. In both cases, it seems that Ni and MgO migrate out of the spinel lattice during the reduction sintering in the surface.^40^ Nonetheless, the Mg-Al spinel crystal size remains constant, around 8 nm, in all cases. On the other hand, the two peaks located at 43.9° and 51.2° in Ni-Fe systems are attributed to the FeNi_3_ alloy. As the Ni content increases, these peaks are sharper pointing out the sintering of this phase with higher Ni percentages. However, its crystal size, shown in Table 2, is much smaller in the case of 2Ni-Fe/MA, 14 nm, while minor Fe/Ni ratios lead to similar crystal sizes around 25 nm. Moreover, it is worth noting the absence of metallic Fe suggesting that, taking into account the alloy stoichiometry as well as the composition of the catalysts, the remaining Fe should have a very small crystal size dispersed on the support nondetectable by XRD.

### 3.2 Reducibility of the Catalysts

**Figure 3:**
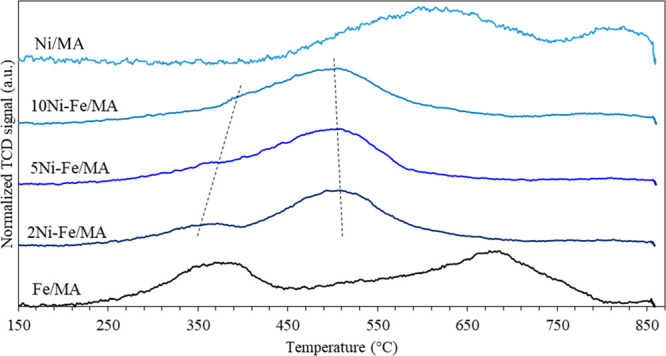
H_2_-TPR profiles of calcined catalysts.

The H_2_ reduction profiles are displayed in Figure 3. Since the support is irreducible (not shown here), the H_2_ consumption of the samples results from NiAl_2_O_4_ and Fe_2_O_3_ reductions. Thus, Ni/MA shows a reduction zone around 600 °C attributed to the well dispersed NiO cluster reduction in addition to another reduction event at 800 °C which is attributed to the NiAl_2_O_4_ spinel reduction.^41^ On the other hand, Fe/MA shows three reduction processes ascribed to the Fe_2_O_3_ reduction (Fe_2_O_3_ → Fe_3_O_4_ → FeO → Fe).^42^ Regarding Ni-Fe systems, they are reduced at lower temperatures than single catalysts most likely due to the H_2_ spillover effect produced by Ni. According to the literature, the first event, located around 360 °C, results from the reduction of NiO and NiFe_2_O_4_ forming Ni along with Fe_3_O_4_. Afterward, the second process, located around 500 °C, is ascribed to the Fe_3_O_4_ reduction to Fe and the Ni-Fe alloy^43^ in fair agreement with our XRD results described previously. Furthermore, as the Fe/Ni ratio decreases, the first reduction event shifts to higher temperatures, while the second reduction event shifts to lower ones due to an increment of Ni-Fe spinel with respect to Fe species. Likewise, the H_2_ consumption, shown in Table 2, increases at lower Fe/Ni due to the increment of the Ni content. In any case, the H_2_ consumed by Ni-Fe systems is greater than that required for the total reduction of Ni species which implies the reduction of Fe species as well. Hence, our TPR and XRD data indicate the presence of dispersed Fe species on the catalyst surface.

### 3.3 Redox Properties

**Figure 4:**
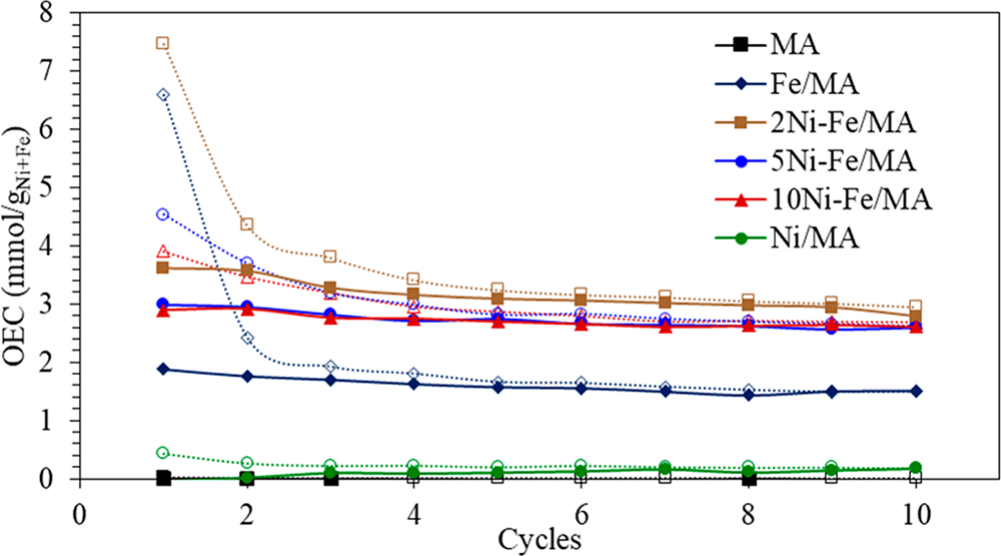
Oxidation (dashed line) and reduction (solid line) cycles of the catalysts at 500 °C: 20 mg of sample, F_T_ = 100 mL/min, 4% CO_2_ for oxidation, and 4% H_2_ for reduction for 20 min.

The RWGS is a redox process, frequently imposing the need to fine-tune the redox behavior of the selected catalysts. The oxygen exchange capacity of the catalysts was measured by thermogravimetric analysis, and the results are recorded in Figure 4. First, it is observed that MA and Ni/MA barely present any OEC since MA is an irreducible support as well as Ni/MA is poorly reduced at 500 °C. On the other hand, the addition of Ni to Fe/MA catalysts boosts the OEC in comparison with undoped Fe/MA due to the H_2_ spillover produced by Ni which facilitates the reduction. However, while a small amount of Ni improves the oxidation and reduction of metals, a large amount of Ni leads to an OEC decrement since an increase of the Ni content promotes alloy formation. Thus, 2Ni-Fe/MA shows the highest OEC among the studied multicomponent catalysts, while 5Ni-Fe/MA and 10Ni-Fe/MA show similar results along the cycles.

### 3.4 Catalytic Activity

**Figure 5:**
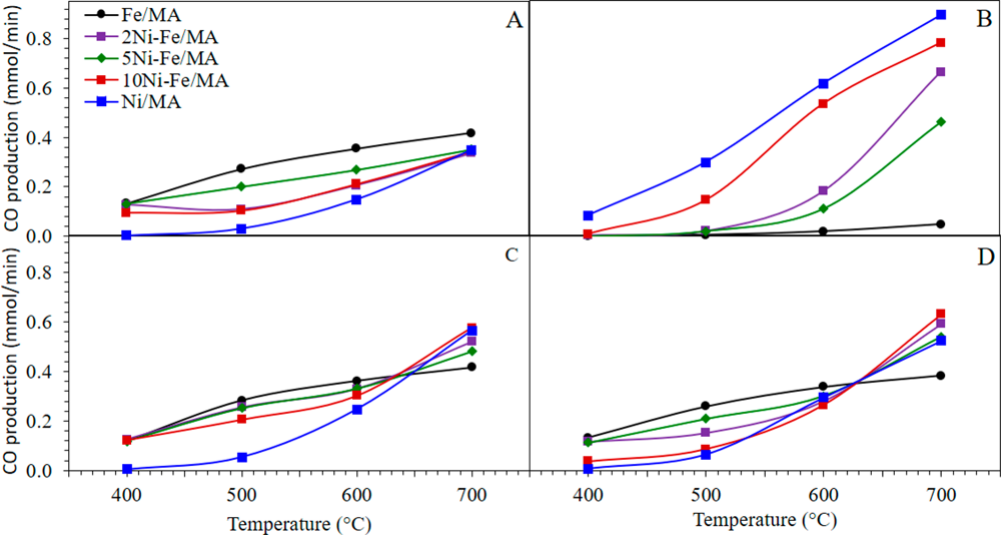
Catalytic activity feeding CO_2_:H_2_:CH_4_: 1:4:0 (A), 1:0:1 (B), 1:4:1 (C), and 1:3:1 (D). F_Total_ = 100 mL/min, 15% CO_2_.

The catalytic activity was, first, measured feeding CO_2_, H_2_, and CH_4_ mixtures in a range of temperatures from 400 to 700 °C, and the CO productivity obtained was collected in Figure 5. Under RWGS conditions (Figure 5A), the Fe/MA catalyst shows higher CO production since it is well-known that Fe-based catalysts favor the RWGS reaction while Ni promotes further hydrogenation to CH_4_.^22^ Moreover, the CO production increases as temperature increases evidencing the endothermicity of the reaction. Thus, Ni/MA presents poor CO production capacity at 400 °C under all studied conditions obtaining mainly CH_4_ (Figure 1). On the contrary, under DRM conditions (Figure 5B), its production increases being that Fe is completely inactive for CH_4_ activation. Regarding Fe/MA decorated with Ni, they show an intermediate activity in both atmospheres. Thus, in comparison with Fe/MA, these catalysts show minor CO production in the absence of CH_4_ at low temperatures. However, the production abruptly rises in the absence of H_2_, especially at 700 °C. Finally, under mixed conditions (Figure 5C,D), although the CO obtained with Fe/MA is higher below 600 °C, the productivity of Ni-Fe systems only decays slightly, especially under RWGS favored conditions (Figure 5C). Nevertheless, at 700 °C, the production of CO is higher with Ni-Fe/MA catalysts being even more productive than Ni/MA in the case of closer DRM conditions. Therefore, the Ni-Fe catalysts enhance the CO production in the presence of both H_2_ and CH_4_ by promoting RWGS and DRM reactions.

**Figure 6:**
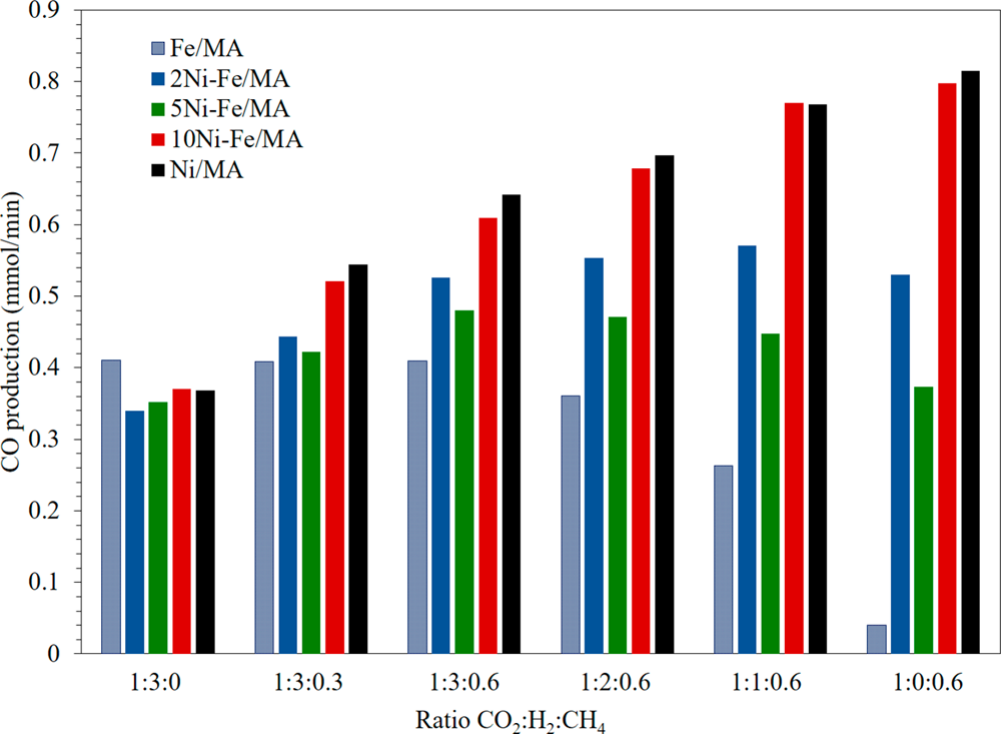
CO production of the catalysts feeding different CO_2_, H_2_, and CH_4_ mixtures at 700 °C. F_Total_ = 100 mL/min.

In order to valorize biomass-gasification derived feedstocks, the catalysts were further evaluated under a variety of CO_2_:H_2_:CH_4_ compositions at 700 °C. Figure 6 displays the CO production obtained in each case being, first, more similar to RWGS conditions (H_2_-rich feeds) and, forward, more similar to DRM conditions (H_2_-poor feeds). It is clearly seen that under more H_2_-rich conditions, Fe/MA produces higher CO, while closer to DRM conditions, i.e., more CH_4_-rich conditions, Ni/MA along with 10Ni-Fe/MA was more productive. Likewise, regarding the Ni-Fe systems, it is observed that the CO production increases as the Ni amount increases in the absence of CH_4_. On the contrary, 2Ni-Fe/MA shows higher productivity than 5Ni-Fe/MA as minor H_2_ is fed.

**Figure 7:**
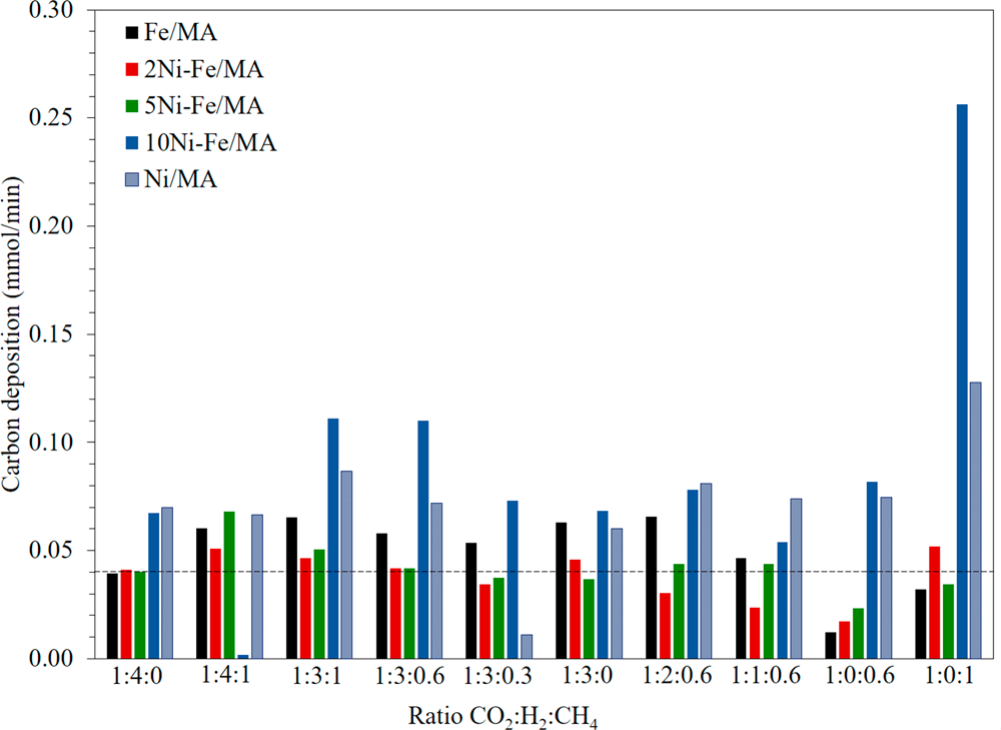
Carbon deposition varying the feed composition at 700 °C: F_Total_ = 100 mL/min, 15% CO_2_.

One important aspect in CH_4_ conversion is the carbon deposition since it compromises the lifetime of the catalysts.^44^ Therefore, in Figure 7, the carbon deposition obtained after 30 min on stream at 700 °C being 0.04 mmol/min the 5% of carbon balance, i.e., the experimental error, is presented. It is clearly seen that the productivity of Ni/MA and 10%Ni-Fe/MA also implies great carbon depositions, especially under DRM conditions. This is a consequence of the DRM reaction mechanism and in particular CH_4_ activation over Ni as evidenced by DFT studies.^45^ Hence, although the CO production is slightly higher using these catalysts, 2Ni-Fe/MA shows an optimal tradeoff in terms of CO production and carbon deposition in the presence of both H_2_ and CH_4_. According to our XRD data, this sample presents the smallest FeNi_3_ alloy particle size being also the sample displaying the highest OEC. The later showcases a clear correlation structure/redox behavior and catalyst performance. The FeNi_3_ alloy is essential in achieving an acceptable balance activity/carbon deposition when such an alloy is well dispersed preserving a small particle size. Moreover, the enhanced OEC evidenced in this sample may hamper carbon deposition by partial oxidation of a solid carbon with a lattice oxygen resulting in a more stable catalyst in terms of cocking. Overall, our 2Ni-Fe/MA system is deemed as a very versatile catalyst for biomass-gasification derived mixture valorization to obtain syngas-rich streams that can be flexibly converted into biofuels and added value chemicals.

## 4 Conclusions

Herein, multicomponent Fe-based catalysts decorated with Ni were evaluated for the syngas production from biomass gasification streams containing CO_2_, H_2_, and CH_4_. The variations on the Ni content affected the catalysts’ structure and their corresponding redox and catalytic behavior. Thus, the structural characterization evidenced the presence of hematite domains dispersed over the MgAl_2_O_4_ spinel support. Besides, all reduced systems exhibited diffraction lines associated with the constitution of FeNi_3_ alloys. The expected optimal performances depicted by Ni-rich systems at high temperatures (especially at 700 °C) toward DRM were accompanied by great carbon depositions compromising the long-term stability of the catalysts. In contrast, Ni-Fe systems show almost no carbon deposition in most of the studied cases being that the 2Ni-Fe/MA is the most promising formulation to avoid coking. The addition of 2 wt % Ni resulted in smaller FeNi_3_ particle sizes and a remarkable OEC. In this sense, the combination of the FeNi_3_ alloy along with enhanced redox features is essential in achieving an optimal balance between catalytic activity and coking resistance.

All in all, this work showcases a catalytic strategy to produce syngas-rich streams from several gasification-derived feedstocks by implementing custom-made Ni-Fe/MA catalysts to promote RWGS and DRM reactions. Our findings pave the way toward the development of flexible biosyngas upgrading processes expanding the horizons of current bioenergy and biofuel production technologies.
